# Role of Pharmacists in COVID-19 Disease: A Jordanian Perspective

**DOI:** 10.1017/dmp.2020.186

**Published:** 2020-06-05

**Authors:** Mariam Abdel Jalil, Mervat M Alsous, Khawla Abu Hammour, Mais M Saleh, Rimal Mousa, Eman A Hammad

**Affiliations:** Department of Biopharmaceutics and Clinical Pharmacy, School of Pharmacy, The University of Jordan, Amman, Jordan; Department of Pharmacy Practice, Faculty of Pharmacy, Yarmouk University, Irbid, Jordan; Department of Pharmaceutics and Pharmaceutical technology, School of Pharmacy, The University of Jordan, Amman, Jordan

**Keywords:** coronavirus, COVID-19, Jordan, knowledge, outbreak, pharmacist

## Abstract

**Objectives:**

The novel 2019 coronavirus outbreak that first appeared in Wuhan has quickly gained global attention, due to its high transmissibility and devastating clinical and economic outcomes. The aim of this study was to assess the possible roles of Jordanian pharmacists in minimizing the stage of community transmission.

**Methods:**

A cross-sectional survey using Google forms targeting Jordanian pharmacists was conducted during March 2020 and distributed electronically by means of social media. Using the survey tool, we measured the pharmacists’ knowledge, the educative activities they perform, and their perceptions regarding undertaking traditional and untraditional roles during the COVID-19 outbreak, as specified by the International Pharmaceutical Federation (FIP). Collected data were analyzed using SPSS version-19.

**Results:**

Jordanian pharmacists (*n* = 449) reported performing various educative activities, and in general, they were knowledgeable about various aspects of the COVID-19 disease (median knowledge score: 20 [range, 13-25]), but certain gaps in knowledge were detected that must be addressed. Pharmacists had positive perceptions about both their traditional and untraditional roles specified by the FIP, the median perceptions score was 4 (range, 1-5).

**Conclusions:**

Jordanian pharmacists can be used to reduce community transmission of the outbreak. However, more actions are required to keep pharmacists knowledgeable with recent disease updates to enable them to perform their tasks effectively during times of crisis.

The novel coronavirus, known as severe acute respiratory syndrome coronavirus-2 (SARS-CoV-2), has been identified as the cause of coronavirus disease 2019 (COVID-19) disease, which was first identified in Wuhan, China, in December 2019.^1^ The novel virus is highly transmittable, and until March 30 has caused 779,575 confirmed cases across 200 countries and territories.^2^ The World Health Organization (WHO) has declared it as a Public Health Emergency of International Concern, pressing countries to take preventive measures to contain this threatening disease.^3^

Jordan confirmed its first case of coronavirus on March 2, 2020. New additional cases continued to appear and the total number of confirmed cases exceeded 270 by the end of March 2020, while fatalities summed up to 5 deaths.^4^ Frontline physicians and nurses are fighting a real battle in hospitals against COVID-19. Beside them are pharmacists who are usually considered the first point of contact people have with the health care system, when they have health concerns, or when they simply need information.^5,6^ In such circumstances, their role is not only limited to ensure an adequate stock of medicine and protective equipment. They also can play an important role in decreasing community transmission by increasing public awareness toward COVID-19 disease among the general Jordanian population, counseling, referring, and avoiding panic. This role is vital in preventing of separability of COVID-19 and overall infection control.^6^

The International Pharmaceutical Federation (FIP) issued a guideline for pharmacists and the pharmacy workforce in February 2020 and was updated several times thereafter. This guidance provides pharmacists with relevant information on the coronavirus outbreak.^6^ The present study aimed to assess the potential roles of pharmacists in public health emergencies by assessing their knowledge, educative activities, and perceptions toward taking traditional and untraditional roles during the outbreak as specified by the FIP.

## METHODS

### Study Design and Ethics

A cross-sectional survey using Google form targeting Jordanian pharmacists was conducted during March 2020 and distributed electronically using social media. The study protocol was approved by the Department of Biopharmaceutics and Clinical Pharmacy of The University of Jordan and the Institutional Review Board of Jordan University Hospital (decision no. 2020/74). The first page of the questionnaire introduced participants to the aims of the study and explained the voluntary nature of participation, then the participants were asked if they would like to take part in the study, and the questionnaire was terminated automatically if participants declined to take part.

### Study Instruments

The survey was developed by the authors of this manuscript in Arabic language, after an extensive review of the literature.^5-7^ The final version of the questionnaire was composed of 5 sections. The first section aimed to collect general information about the participants, such as age and gender. The second section was divided into 2 parts: the first part was the knowledge scale, which was divided into 5 domains as shown in Table 2; the second part enquired about the awareness of pharmacists regarding the presence of FIP guidance. The third section aimed to assess the perceptions of pharmacists regarding their expected traditional and untraditional roles during the COVID-19 outbreak as identified by the FIP. The fourth section assessed the sources of information pharmacists use and the degree to which they trust these sources. The final section was asking pharmacists to report the frequency of selected activities performed to educate the public about the COVID-19 outbreak. The questionnaire was reviewed by the authors and then subjected to pilot testing using a small group of participants, which resulted in several minor amendments.

**TABLE 1:** General Characteristics of the Included Pharmacists, n = 449

Characteristic	N (%)
Age (year):	
18-25	151 (33.6%)
26-35	169 (37.6%)
36-45	92 (20.5%)
46-55	34 (7.6%)
≥ 56	3 (0.7%)
Gender:	
Male	367 (81.7%)
Female	82 (18.3%)
Degree	
Diploma	29 (6.5%)
BSc	312 (69.5%)
Master or PhD	108 (24.0%)
Pharmaceutical field:	
Hospital pharmacists or clinical pharmacists	85 (18.9%)
Community pharmacists	144 (32.1%)
Academia and research	76 (16.7%)
Other^a^	79 (17.8%)
Does not work currently	65 (14.5%)
Years of experience	
0-5	257 (57.2%)
6-10	85 (18.9%)
11-15	49 (10.9%)
16-20	28 (6.3%)
21-25	17 (3.8%)
>25	13 (2.9%)

a Other fields included for example pharmacists working in the pharmaceutical industry and pharmaceutical marketing.

**TABLE 2:** Overview of the Knowledge Section

Part1: Knowledge Scale			
Domain		% Answered Correctly	Average Subscale Score (SD)
**A. General Knowledge (Maximum Score 5)**			
1	The novel coronavirus was discovered in the Chinese city Wuhan at the end of 2019. *Correct *Incorrect *I do not know	95.8%	**4.6 (0.62)**
2	The official name that has been announced for the virus responsible for COVID-19 disease is severe acute respiratory syndrome coronavirus 2. *Correct *Incorrect *I do not know	79.5%	
3	COVID-19 disease infects only the elderly and those with chronic diseases. *Correct *Incorrect *I do not know	94.9%	
4	Individuals most likely to die from COVID-19 disease are: *Elderly *neonates *children * I do not know	97.1%	
5	The incubation period for COVI-19 disease in days is: *1-4 *5-10 *2-14 *15-55 *I do not know	90.2%	
**B. Knowledge Regarding Symptoms and Modes of Transmission (Maximum Score 4)**			
1	The main symptoms of infection with the coronavirus are fever, cough, shortness of breath, and general fatigue. *Correct *Incorrect *I do not know	100%	3.4 (0.53)
2	Possible ways through which the virus can spread include: *Droplets *air *feces *all *I do not know	96.9%	
3	What we know so far is that pets can spread the virus to humans. *Correct *Incorrect *I do not know	40.1%	
4	COVID-19 virus can be transmitted from an infected person that does not show symptoms. *Correct *Incorrect *I do not know	98.2%	
**C. Knowledge Regarding Protective Measures and Treatments (Maximum Score 8)**			
1	In general, wearing a medical mask is required for workers in the health sector and those who are in contact with patients. *Correct *Incorrect *I do not know	98.0%	5.6 (0.9)
2	Use an N95 respirator alone is enough to provide an adequate level of protection from the risk of infection with the virus when providing health care for a confirmed case. *Correct *Incorrect *I do not know	76.6%	
3	Can the flu vaccine be used to prevent infection from the coronavirus? *Yes *No *I do not know	90.4%	
4-6	Which of the following is an effective means in preventing the spread of the coronavirus?		
	a) *Washing hands with water and soap frequently*	100%	
	b) Staying half a meter away from an infected person	27.6%	
	c) *Refrain from touching the mouth, nose, and eyes before washing hands*	85.2%	
7	In hospitals and pharmacies, all surfaces that may be contaminated with the virus must be sterilized. All of the following can be used in the sterilization process, with the exception for: * Ethanol (75%) * Chlorhexidine * Peracetic acid * Chloroform * I do not know	6.5%	
8	Are there FDA approved antibiotics for treating COVID-19 patients? * Yes *No *I do not know	65.9%	
**D. Misinformation Associated With COVID-19 (Maximum Score 4)**			
**1**	Drinking anise tea can help in preventing the infection with the coronavirus. *Correct *Incorrect *I do not know	63.7%	2.5 (1.4)
**2**	Eating garlic helps in preventing the infection with the coronavirus. * Correct *Incorrect *I do not know	65.0%	
**3**	Rinsing your nose regularly with salty water helps in preventing the infection with the coronavirus. * Correct *Incorrect *I do not know	45.0%	
**4**	Applying sesame oil prevents the entry of the virus causing COVID-19 disease. * Correct *Incorrect *I do not know	77.1%	
**E. Referral of Suspected COVID-19 Cases (Maximum Score 4)**			
**For Each of the Following Cases Indicate if the Patient Should Be Referred to a Suitable Health Care Facility for Further Tests:**			
**1**	Fever or shortness of breath without history of travel or interaction with COVID19 cases.	55.0%	3.5 (0.56)
**2**	Fever, coughing, and shortness of breath, as well as a history that includes travel from Hubei Province in China within 14 days of symptoms onset.	99.3%	
**3**	Fever, coughing, and shortness of breath, in addition to a history of close contact or touching a patient whose disease has been confirmed within 14 days of symptoms onset.	98.8%	
**4**	Fever, coughing, and shortness of breath that requires hospitalization and a history of travel from mainland China within 14 days of symptoms onset.	98.2%	
**Part 2: Awareness Regarding the Availability of the FIP COVID-19 Guidelines for Pharmacists and the Pharmacy Workforce**			
	Do you know that the International Pharmaceutical Federation (FIP) has issued a guidance for COVID-19 related to pharmacists and the pharmacy workforce?		
	Yes, and I have read it	18.9%	
	Yes, but I did not have a chance to read it	24.7%	
	No, I don’t know it exists	56.4%	
	Correct answers are in *italics*.		
	Based on the initial FIP guidance (05/02/2020) case 2-4 should be referred.		

### Statistical Analysis

#### Sample Size Calculation and Statistical Analysis

Assuming an unlimited population, 95% confidence level, and a 5% margin of error, the minimum required sample size is 385 participants.^8^ Collected data were analyzed by means of SPSS version-19. Percentages were used to describe categorical data (pharmacists’ characteristics). As for continuous data, the Shapiro-Wilk test was used to test the normality of continuous variables. Because the results of this test indicated that all continuous variables were nonparametric, the Mann-Whitney U test was used to compare continuous variables between groups. Categorical variables were gender, field of pharmaceutical work (hospital and community pharmacists versus other fields of pharmaceutical work or unemployment), or years of experience (greater or less than 5 years of experience). Statistical significance was set to *P* < 0.05.

#### Excluding Careless Responses

A dichotomous self-reported single item indicator was used to exclude careless responses.^9^ At the end of the questionnaire, participants were asked if their data should be included in the analysis. Any participant that answered this question with a “No” was excluded from the study.

## RESULTS

### Participants’ Characteristics

During the study period, we received responses from a total of 578 participants, of those 129 responses were excluded (4 declined to take part in the study after reading the consent form, 69 reported that they were still students, 40 reported not being pharmacists, and 16 pharmacists reported careless responses); thus, the present analysis included responses from 449 pharmacists, the characteristics of the included participants are presented in Table 1.

### Pharmacists’ Knowledge

The overall median knowledge score for the included pharmacists was 20 (range, 13-25); there was no difference in knowledge score based on age, gender, or field of pharmaceutical work. Results regarding the knowledge section are portrayed in Table 2. Each correct answer was given a mark of 1, incorrect answers or an answer of “I do not know” was given a score of 0.

### Sources of Information

The most common sources of information used by the respondents were global websites such as the websites of the World Health organization (WHO), Centers for Disease Control and prevention (CDC), or the FIP as reported by 80.6% of respondents, followed by the Jordanian television and official sites (61.2%), physicians (41.6%), unofficial news sites (26.3%), and friends, relatives, and colleagues (15.1%).

Complete trust in official websites such as the CDC, WHO, FIP was reported by 88.6% of the recruited pharmacists; approximately one-third of those pharmacists also reported completely trusting Jordanian television and official websites (35.4%) and physicians (34.1%). Less reliable sources of information included unofficial websites, as they were completely trusted by only 2.2% of pharmacists, in addition to friends, relatives, and colleagues as only 1.8% of respondents stated that they trusted them completely.

### Pharmacists Perceptions About Their Role in Public Health Emergencies

The perceptions of pharmacists about their traditional and untraditional roles (referral) during COVID-19 disease were positive, as shown in Table 3. Furthermore, there was no significant difference in perceptions scores between pharmacists based on gender, field of pharmaceutical work, or years of experience.

**TABLE 3:** Perceptions of Pharmacists About Their Roles During COVID-19 Outbreak

**Statement**	**Average Perceptions Score** ^a^	**%Positive Perceptions** ^b^
Community pharmacists can promote public education about COVID-19 disease.	4.74	99.6%
Community pharmacists can enhance COVID-19 disease preventive behaviors in the community.	4.75	99.8%
*Community pharmacists cannot enhance infection control efforts in the event of an outbreak.*	3.37	76.4%
*Community pharmacists cannot refer suspected cases in a timely and safe manner to the appropriate health care facilities.*	3.32	76.4%
Community pharmacists have an important role and an ethical responsibility in providing masks to the local community at reasonable prices.	4.69	99.1%
Community pharmacists have an important role and ethical responsibility in providing sterilization tools to the local community at reasonable prices.	4.72	99.6%
Hospital pharmacists have an important role in storing appropriate quantities of medicines for treating COVID-19 disease (if available) and other medical products to secure demand.	4.35	95.3%
*Hospital pharmacists have no important role in providing patient care and support during periods of the disease outbreak.*	3.54	80.5%
*Hospital pharmacists do not have an important role in ensuring the prevention and control of COVID-19 infection in hospitals.*	3.61	82.8%
Hospital pharmacists have an important role in providing information regarding COVID-19 disease.	4.44	95.6%

a The perceptions of pharmacists were measured using a 5-item Likert scale. [strongly disagree, disagree, neutral, agree, and strongly agree]. The items were scored as; 1, 2, 3, 4, 5 correspondently for all sentences apart of those in *Italics*, where the scores were reversed.

b %Positive perceptions were calculated by summing the frequency of pharmacists that answered “strongly agree,” “agree,” and “neutral” apart from statements in *italics,* where % positive perceptions were calculated by summing the frequencies of “strongly disagree,” “disagree,” and “neutral.”

### Jordanian Pharmacists’ Activities During Public Health Emergencies

During the COVID-19 outbreak in Jordan, pharmacists were asked to report how frequently they performed various activities in the preceding fortnight, the results are portrayed in Table 4.

**TABLE 4:** Frequency of Performed Activities in the Preceding Fortnight

Activity	0	1-5	6-10	11-15	16-20	21-25	26-30	>30
Educating citizens about the nature of the disease in general.	6.2%	35.0%	24.7%	16.5%	3.6%	1.3%	1.3%	11.4%
Educating citizens about the symptoms of the disease.	7.1%	31.2%	28.5%	16.3%	2.9%	1.3%	2.7%	10.0%
Educating citizens about the ways it spreads.	6.0%	29.4%	29.8%	17.4%	2.9%	1.1%	2.0%	11.4%
Educating citizens about ways to prevent it.	6.0%	28.5%	27.2%	19.4%	2.9%	1.6%	2.2%	12.2%
Distributing awareness brochures about COVID-19 disease.	69.7%	15.4%	6.0%	4.5%	0.4%	1.3%	0.0%	2.7%

More elaborate activities were also performed since the beginning of 2020, such as conducting seminars to a minimum of 5 people at least once as reported by 25.4% of pharmacists. Preparing educational videos related to the COVID-19 disease at least once as reported by 22.5% of pharmacists, and preparing alcohol-based disinfecting preparations in the pharmacy, which was conducted at least once by 17.4% of responding pharmacists.

## DISCUSSION

During public health emergencies, pharmacies and pharmacists’ services need to be continued and are likely to be extended beyond their traditional scope of practice.^10,11^ A recent study published by Amariles et al.^12^ highlighted how pharmacists can have a crucial role in minimizing the stage of COVID-19 community transmission by means of patient education, appropriate detection, referral, and management of suspected cases.

To evaluate the ability of pharmacists to act as educators, we assessed their knowledge on various aspects of COVID-19 disease. The results showed that they performed well in terms of general aspects; however, there were some gaps in knowledge in terms of disease transmission; for instance, 43.2% of pharmacists acknowledged that pets can transmit the disease to humans. During the survey period and to date, there is no evidence that pets have a significant role in transmitting the COVID-19 disease to humans.^13^

Because the virus is mainly spread through person-to-person contact, it is recommended to avoid close contact by leaving a distance of 2 meters away from any sick person. This information needs be highlighted to Jordanian pharmacists, because as many as 72.4% stated that staying half a meter away from a COVID-19 patient is effective in preventing the spread of the virus. Another important topic that needs to be reinforced is disinfecting management. Any area within the pharmacy environment can be easily contaminated and, hence, must be disinfected. The following disinfectants could effectively inactivate SARS-CoV-2: 75% ethanol, chlorine-containing disinfectants, peracetic acid, and chloroform but not chlorhexidine.^6^ Unfortunately, in our sample only 6.5% of pharmacists answered correctly the question relating to SARS-CoV-2 disinfectants.

Previous research studies have proved that herbal medicine is widely used by Jordanian cancer patients and patients with chronic diseases.^14-16^ Despite the availability of highly sophisticated modern medicine in Jordan, the wide use of herbal medicine could be attributed to cultural reasons. This could explain why approximately one-third of pharmacists were not aware that drinking anise tea and eating garlic have not been proven to prevent infection with COVID-19. Surprisingly, even though 80.6% of pharmacists stated that they use the WHO and CDC website as a source of information, only 45% of pharmacists were aware that rinsing the nose regularly with saline does not help in preventing the infection with the coronavirus. The usefulness of this method was widely circulated through various social media platforms, despite being refuted by the WHO.^17^ It is expected that every health emergency will be surrounded by a tsunami of misinformation.^18^ Nevertheless, pharmacists must have enough knowledge and resources to be able to judge the credibility of health-related information.

Based on the initial guidance produce by the FIP for pharmacists and pharmacy workforce (February-2020), and the Jordanian emergency guidelines for the management of COVID-19 (March-2020), any individual with fever or respiratory symptoms and either a history of travel to China (or other infected areas) or having contact with COVID-19 confirmed cases should be referred to appropriate health care facilities for further testing. Based on this, almost all Jordanian pharmacists answered 3 of 4 cases correctly as shown in Table 2. Given the fact that the disease had spread at a tremendous speed, the FIP recommendations changed in the following month and pharmacists should advise only high-risk individuals in the above scenarios to contact the emergency department for further testing.

Overall, pharmacists had positive perceptions about their traditional and untraditional roles during the COVID-19 outbreak as reported by the FIP. Furthermore, they frequently educated the public about the disease, its symptoms, how it is spread and how to prevent it, with only up to 7.2% of pharmacists reporting not doing any of these activities in the preceding fortnight. A smaller proportion of Jordanian pharmacists reported being involved in more advanced activities since the beginning of the outbreak even before it spread to Jordan, such activities include preparing educative videos, giving seminars about the disease, and preparing alcohol-based disinfecting preparations in pharmacies.

The present study has several limitations, which include the possibility of recall bias, as 5 questions asked pharmacists to recall the activities they performed over the preceding 2 weeks, while 3 questions requested pharmacists to recall activities performed up to 3 months before taking the survey. Moreover, the response rate could not be calculated as the questionnaire was distributed electronically. Another limitation was that a large proportion of respondents were young pharmacists (≤35 years), which could be explained by the lack of interest of older pharmacists (>35 years) in taking part or lack of access to social platforms. The higher percentage of female participants in the present study could also indicate the lack of interest of male pharmacists in answering our questionnaire. Nevertheless, it is important to highlight that the largest proportion of pharmacists in Jordan are females. For instance, up to February 2019, females were 64.4% of all registered pharmacists according to the Jordan pharmacists association records.^19^

## CONCLUSIONS

The present study assessed the possible role of pharmacists in reducing community transmission of COVID-19 by assessing their knowledge and various educative activities they perform. In addition to their perceptions regarding undertaking traditional and untraditional roles, as specified by the FIP. Although Jordanian pharmacists were knowledgeable about various aspects of the COVID-19 disease, certain gaps in knowledge existed, especially in parts relating to disinfection reagents and certain common cultural and social media misinformation.

Traditional roles of pharmacists, such as medication-related activities, need to be extended in cases of emergencies; such extended activities include triage and referral activities. Despite the positive perceptions about all roles specified by the FIP, it is important to pinpoint that, during emergencies, both traditional and untraditional roles become difficult to implement. Thus, an effective general framework of action is needed in times of crises to enhance pharmacists’ knowledge and their ability to work under pressure in such difficult times, although the form and content of these frameworks can be challenging, as it is the feature of crises to come in an unpredicted form and magnitude.^10,11^ It is in our opinion, effective teamwork and communication channels and the presence of a formal Jordanian pharmaceutical platform that tailors information to pharmacists and organizes tasks can help them in performing both their traditional and untraditional roles.
